# Social carry‐over effects underpin trans‐seasonally linked structure in a wild bird population

**DOI:** 10.1111/ele.12669

**Published:** 2016-09-13

**Authors:** Josh A. Firth, Ben C. Sheldon

**Affiliations:** ^1^ Edward Grey InstituteDepartment of ZoologyUniversity of OxfordOxfordOX1 3PSUK

**Keywords:** Carry‐over effects, habitat selection, social networks, social relationships, spatial structure, territory choice

## Abstract

Spatial structure underpins numerous population processes by determining the environment individuals' experience and which other individuals they encounter. Yet, how the social landscape influences individuals' spatial decisions remains largely unexplored. Wild great tits (*Parus major*) form freely moving winter flocks, but choose a single location to establish a breeding territory over the spring. We demonstrate that individuals' winter social associations carry‐over into their subsequent spatial decisions, as individuals breed nearer to those they were most associated with during winter. Further, they also form territory boundaries with their closest winter associates, irrespective of breeding distance. These findings were consistent across years, and among all demographic classes, suggesting that such social carry‐over effects may be general. Thus, prior social structure can shape the spatial proximity, and fine‐scale arrangement, of breeding individuals. In this way, social networks can influence a wide range of processes linked to individuals' breeding locations, including other social interactions themselves.

## Introduction

The spatial structure within populations is an important aspect of many biological processes (Tilman & Kareiva 1997; Lion & van Baalen 2008). For example, fine‐scale spatial positions of individuals within heterogeneous landscapes can govern the environmentally imposed selection pressures they face (Blondel *et al*. 1999; Richardson *et al*. 2014; Hadfield 2016). Further, the heritability of phenotypic traits, and thus responses to selection, can also vary over small scales (Garant *et al*. 2005), and therefore spatial structure can have consequences for adaptation over time.

An individual's spatial position also determines which other individuals it encounters, thereby influencing its social environment (Crook 1970). Therefore, where individuals are spatially located shapes processes dependent on social interactions, such as cooperation (Nowak & May 1992; Lion & van Baalen 2008) and mating (Emlen & Oring 1977). For instance, individuals inhabiting areas where they encounter conspecifics more often may thus experience a higher intensity of sexual selection (Taff *et al*. 2013), and males may increase their reproductive success if their local competitors are systematically less‐competitive individuals (Oh & Badyaev 2010; Farine & Sheldon 2015).

Despite its broad significance for a wide range of ecological processes, elucidating the drivers of individuals' spatial distributions with respect to one another is challenging. It has previously been recognised that individuals may base themselves in areas that best suit their own attributes (Clobert *et al*. 2009; Wey *et al*. 2015) and base their decisions on external environmental features, such as habitat quality and predation risk (Cody 1985), and local population factors, such as mating opportunities and competition (Clobert *et al*. 2001, 2004; Bowler & Benton 2005). Yet, how social associations between individuals (the social landscape) shapes where they locate themselves remains largely unknown (Shizuka *et al*. 2014; Wey *et al*. 2015).

Individuals may benefit from positioning themselves near familiar individuals, as close social associates offer potential benefits, such as access to information (Lachlan *et al*. 1998; Aplin *et al*. 2012; Firth *et al*. 2016), reduced aggression (Temeles 1994) and increased reciprocation of cooperation (Croft *et al*. 2006; Grabowska‐Zhang *et al*. 2012a; Carter & Wilkinson 2013). This may result in fitness benefits of holding strong social connections to local individuals; previous examples include increased growth in fish species (Hojesjo *et al*. 1998; Seppa *et al*. 2001) and improved reproductive output of birds (Beletsky & Orians 1989; Grabowska‐Zhang *et al*. 2012b). In this way, selection may act to shape individuals' choice of spatial location around their close social associates. Elucidating this process would aid in understanding both the importance of maintaining social associations, as well as how social associations can underpin future fine‐scale spatial positioning and the resultant consequences of this.

As with many other bird species, great tits (*Parus major*) choose a single spatial location for nesting, egg laying, incubation and raising their brood over the breeding season. Breeding positioning is also known to profoundly influence various ecological processes (Chalfoun & Schmidt 2012), and previous research within our study population has demonstrated relationships with mating, cooperative interactions, response to selection, phenology and timing of breeding (Garant *et al*. 2005; Grabowska‐Zhang *et al*. 2012a; Patrick *et al*. 2012; Cole *et al*. 2015; Firth *et al*. 2015a; Hinks *et al*. 2015). Yet, as with other ecological systems, the underlying forces driving fine‐scale spatial structure remain largely unexplored. Here, we monitored winter social associations among wild great tits, as individuals join flocks and move relatively freely around the woodland. We assess how these social networks, formed when individuals are not required to base themselves at a fixed position, carry‐over into shaping the set locations at which they breed during the spring. Hence, we determine how prior social structure relates to future spatial positioning, to understand the potential importance of previous social associations and the possible implications for processes governed by population spatial structure.

## Material and Methods

### Study system

The work was carried out in Wytham Woods, Oxford, UK (51°46′ N, 1°20′ W), on a great tit population that has been monitored using standardised protocols since the 1960s (Perrins 1965). The great tits breed almost exclusively in the nestboxes located in 1020 fixed positions with known GPS coordinates throughout the woodland (Wilkin *et al*. 2006, 2007). Most breeding birds (> 98%) are only recorded at one nestbox in any single year, and their breeding attempt consists of nest building, egg laying, incubation and offspring rearing, which spans the breeding season from April to July. During this time, nestboxes are visited regularly to record breeding attempts and identify adults (between days 6 and 14 of the nestling phase) and mark nestlings (on day 15) with a unique BTO (British Trust for Ornithology) metal leg ring and record standard morphometric information.

Throughout the winter season (September–March), great tits aggregate to form roving feeding flocks (Hinde 1952) that show considerable turn‐over as different birds leave and join frequently, as flocks display high ‘fission‐fusion’ dynamics (Farine *et al*. 2015). Since 2007, plastic leg rings containing unique RFID‐microchips have been fitted to all great tits captured either during breeding or through winter mist‐netting. This results in ~ 90% of the population being tagged (Aplin *et al*. 2013). The RFID tags allow detection of times and locations of individuals' occurrence at sunflower feeding stations equipped with two RFID‐antennae (Dorset ID, Aalten, The Netherlands) placed in 65 locations in a stratified grid. In winters beginning 2011, 2012, and 2013, the feeders opened every weekend from December until the end of February (13 weekends), scanning for RFID tags from visiting birds from pre‐dawn to post‐dusk.

### Winter population social structure

The feeding stations' detections of RFID tagged birds generate a spatio‐temporal data‐stream consisting of bursts of activity as flocks arrive and feed. Each detection was then statistically assigned to the flock, or ‘gathering event’, it most likely belonged to using a machine‐learning algorithm (Psorakis *et al*. 2012). This method is robust, effective for determining flock co‐memberships, and performs significantly better than alternative methods such as using arbitrary time windows to define co‐occurrence (Psorakis *et al*. 2015). From the flock co‐memberships, social networks (association matrices) were constructed in R 3.2.2 (R Core Team 2015) using the Simple Ratio Index (Cairns & Schwager 1987) defined here as follows: $S_{AB}=\frac{x}{x+y_{AB}+y_{A}+y_{B}}$, where $S_{AB}$ is the social association between bird *A* and *B*,* x* is the number of times they co‐occurred in the same flock and $y_{AB}$ is the number of times they were both detected at the same time but not together. $y_{A}$ is the number of flocks that *A* occurred in without *B* over the time period both were known to be in the system, and $y_{B}$ is the number of flocks that *B* occurred in without *A* over this period.

Social networks were created on a yearly (whole winter) basis, to measure the propensity of dyads to associate, as well as per weekend (i.e. per sampling period) to quantify co‐occurrence during weekly intervals. We used non‐directional weighted networks throughout our analyses that, along with the extensive sampling, reduces the limitations arising from employing a ‘gambit of the group’ approach (Franks *et al*. 2010). Social networks were built using all individuals, but in further analysis, we only considered breeding individuals. While mated pairs were included in the analysis, we excluded the dyadic association between partners, as these have strong social associations (Psorakis *et al*. 2012; Firth *et al*. 2015b) and share a breeding location. Due to high population turnover and individual movement, kin structure was very weak (< 1.5% of winter social connections were between first‐order relatives); therefore, relatedness was not considered in any of the analysis.

#### Carry‐over of winter social networks into breeding spatial proximity

We aimed to assess whether increased social associations between birds during the winter related to closer spatial proximity of their breeding positions in the subsequent spring. We assessed spatial proximity in two ways. First, we calculated Euclidean distance between each nestbox location and used the reciprocal of this as a measure of proximity. Second, we determined the ranked proximity between each box, including only nests containing eggs (i.e. occupied boxes), to establish the relative closeness of the surrounding individuals given the local breeding density. We then examined the ability of the winter social association matrix to predict the subsequent breeding proximity matrix.

As with any social network analysis, spatial and social effects are likely to be related. An individual's choice of winter spatial location will influence both which birds they associate with, as well as their subsequent breeding location. In order to separate social and spatial effects, following previous work (Shizuka *et al*. 2014; Firth & Sheldon 2015), we used two, complementary, approaches, namely (1) null models and (2) Multiple Regression Quadratic Assignment Procedure (MRQAP). In this way, we assess how dynamic winter social preferences *within* individuals' winter spatial locations predict subsequent spatial positioning of individuals' breeding decisions. Further, we also examined (3) how individuals' characteristics may influence this relationship. As social preferences themselves may potentially influence winter spatial decisions, both methods of controlling for winter spatial occurrence represent a conservative approach to examining the influence of social associations (Shizuka *et al*. 2014; Aplin *et al*. 2015).

##### Spatial null model

For each year separately, we used mantel tests (Smouse *et al*. 1986) to determine the correlation between the previous winter's social association matrix and the subsequent breeding proximity matrix. We then determined how this compared to that expected from individuals' winter spatial locations alone. Specifically, if individuals remain and breed at the same location as that they occupy at the end of the winter, this may cause them to breed close to others that also occurred there during the winter. Therefore, we used a null model that controlled for this winter spatial process (see Supplementary Information). In each permutation of the null model, each individual's social network position was randomly reassigned to another individual that had also been observed in the same final winter location as them (Fig. S1). Following this reassignment, the correlation between this permuted social network and the observed breeding proximity matrix was recalculated. Thus, each permutation maintained the overall social network structure and individuals' winter spatial locations but reassigned their dyadic social associations within this. We repeated the permutation procedure 1000 times, and hence derived the distribution of the correlation expected between winter social associations and breeding proximity given individuals' winter spatial locations alone. By comparing this empirically generated expected distribution to the observed correlation between the social network and breeding proximity matrix, we were able to infer the additional influence of dyadic social associations on breeding proximity, over and above that expected from winter spatial structure. Specifically, if the observed test statistic (in this case, Mantel test correlation) fell outside the 95% range of those generated from the permutations, this indicated a statistically significant effect.

##### Matrix regression models

We also used MRQAP (Dekker *et al*. 2007) to quantify how winter social associations related to subsequent breeding proximity given winter spatial factors. This approach has previously been used to separate the effects of winter social structure and winter spatial range overlap (Shizuka *et al*. 2014; Firth & Sheldon 2015), as it allows a single‐dependent matrix to be regressed against multiple ‘predictor’ matrices. Therefore, we examined how subsequent breeding proximity was simultaneously predicted by the winter social association matrix and the winter spatial overlap matrix. The winter spatial overlap was based on the activity of each individual at each feeding station and ranged from 0 to 1, where dyads for which spatial activity patterns fully overlapped would score 1, whilst those that never occurred in the same place would score 0. Specifically, winter spatial overlap for each dyad was calculated as the sum, over all locations, of the minimum proportion of activity that either member spent at each location (see Supplementary Information for details).

##### Individual traits

We considered that the relationship between winter social associations and subsequent breeding proximity might differ depending on the type of individuals considered. In all cases, we used a general linear mixed model (GLMM) structure, which set the dependent variable as the subsequent breeding proximity between each dyad and fitted winter social association strength, year and other effects specific to the question of interest (see below) as predictor variables. Random effects of both individuals' identities and their nestboxes were also included. For each model, to determine the effect of winter association strength on subsequent breeding proximity in comparison to that expected from individuals' winter spatial locations, we carried out the GLMM 1000 times, but each time using a set of dyadic winter social association strength values generated from the spatial null model. The distribution of coefficients derived from these models illustrates that expected by individuals' winter spatial locations alone. Therefore, if the coefficient from the observed data fell outside the 95% range of this distribution, the effect was judged statistically significantly different to that expected from individuals' winter spatial locations. Three types of individual characteristic were considered.

First, as breeding proximity relates to mating opportunities (Patrick *et al*. 2012; Firth *et al*. 2015a), whether a dyad is of the same or opposite sex may influence how prior social association relates to subsequent breeding proximity. Therefore, we additionally included a factor indicating whether each dyad was same sex or opposite sex, along with its interaction with winter social association.

Second, adult birds' breeding proximity may be influenced by their breeding locations in the previous year, particularly if individuals remain in the same locations over years. Therefore, we assessed whether winter social associations remained an important predictor variable of subsequent breeding proximity, even given prior breeding positions, by including previous breeding proximity as a predictor variable in the model (which considered only adults that had bred the previous year).

Finally, individuals that were observed at only a single foraging location throughout the winter might be expected to hold strong social associations to others who remained there over winter, as well as to subsequently breed there. Therefore, we also carried out the GLMM but only including dyadic associations between individuals occurring at the same single location as one another all winter. This allowed us to directly assess whether individuals choose to breed closer to the ones those were most associated with even within a single winter location.

#### Carry‐over of winter social networks into breeding spatial arrangements

The spatial arrangement of individuals' territories (i.e. which individuals share boundaries) has been shown to be important in this population (Wilkin *et al*. 2006, 2007; Grabowska‐Zhang *et al*. 2012b; Hinks *et al*. 2015). Indeed, even given dyads of equal breeding proximity to one another, those that share territory boundaries (defined here as ‘neighbours’) may be more likely to encounter/interact with each other during the breeding season than those who do not (defined as ‘non‐neighbours’). Therefore, we tested whether, controlling for breeding proximity, breeding individuals were more likely to share territory boundaries with those that they had closely associated with in the preceding winter.

We created Thiessen polygons (voronoi diagrams) around each occupied nestbox that encompassed the area closer to it than to any other occupied box (Aurenhammer 1991). Territories created using this method correlate highly with mapped territory size and neighbours, and are known to be biologically meaningful in terms of local population density and breeding success (Adams 2001; Wilkin *et al*. 2006; Valcu & Kempenaers 2008; Grabowska‐Zhang *et al*. 2012a; Schlicht *et al*. 2014). In this way, we defined which individuals were ‘neighbours’ (i.e. shared a territory boundary), and therefore may be more likely to interact with each other than indicated by proximity alone.

On average, however, neighbours are also more proximate than non‐neighbours. Therefore, for each individual, we found all other individuals that fell within the boundaries of an annulus set by distances to their closest non‐neighbour and their furthest neighbour (Fig. S2). This yields the set of individuals that were within a distance range to be potentially either neighbours or non‐neighbours for each focal individual. Using only these dyads, we used a GLMM to determine whether individuals were more strongly associated in winter with their subsequent neighbours than with their non‐neighbours, even after controlling for their breeding proximity. We set each individual's winter social associations to other individuals whose breeding locations fell within this annulus as the dependent variable and fitted fixed effects of breeding proximity, a binary indicator of whether or not the individuals were breeding neighbours, and year. Random effects of both individuals' identities and their nestboxes were also included. By including both of these breeding spatial terms within the model, we were able to examine whether neighbours had stronger prior winter social associations than non‐neighbours of an equal breeding proximity. Again, we repeated this GLMM for each permuted data set, so in each case, the observed social associations between an individual and those that fell within the specified boundary were replaced with the winter social associations generated from the spatial null model. We then the compared the range of the resultant coefficients of the binary ‘neighbour indicator’ from these models to the coefficient calculated from the observed data.

Finally, we calculated individuals' mean social associations to their future breeding neighbours from each of the separate weekly social networks and examined whether individuals' social associations to their future breeding neighbours changed through the preceding winter. We then compared the observed value of individuals' social associations to their future neighbours to the values generated from the spatial null model for each weekly social network.

## Results

The winter social networks consisted of 1092, 721, and 809 individuals (in 2012, 2013, and 2014. respectively) observed over large numbers of flocks (2012 = 73 364; 2013 = 65 095; 2014 = 64 629). The majority of breeding birds had been observed in the previous winter network (~ 75%). Breeding individuals were recorded (on average: mean ± SE), on 10.5 ± 0.1 weekends at 3.5 ± 0.1 different feeding sites, with 13.9 ± 0.5 movements between sites over the 3‐month winter period (December–February).

#### Winter social networks carry‐over into breeding spatial proximity

**Figure 1:**
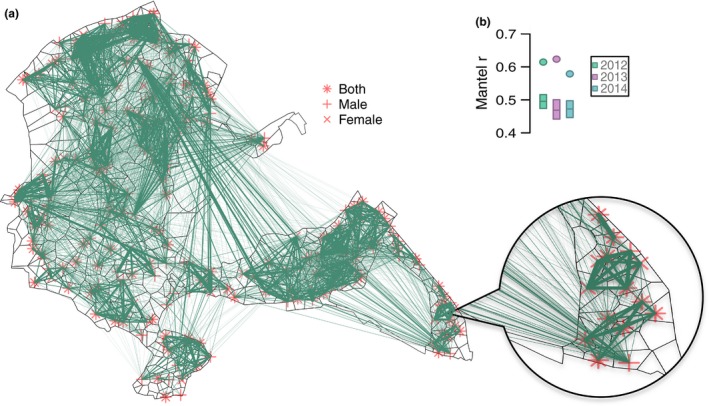
a) Illustrative breeding positions and winter social network of great tits from 1 year (2012) in Wytham Woods. Black dividing lines show the inferred territory boundaries around each individuals breeding location. Points show breeding sites of birds recorded in winter social network (star = both parents, cross = male, ‘X’ = female). Adjoining lines represent social associations recorded in the previous winter, and line thickness illustrates strength of association (mean strength is displayed for boxes where both parents were included). A section has been enlarged for clarity. b) Mantel r test statistic assessing the relationship between winter social networks and subsequent breeding proximity matrices. Circles show the observed statistic and boxes show the 95% range of statistics calculated from the spatial null model (mid‐lines illustrate mean of these). Proximity is calculated from Euclidean distance. See Fig. S5 for ranked proximity and mantel tests for each class of individuals separately.

Individuals generally bred closer to birds they had held stronger social associations with during the previous winter (Fig. 1a; Fig. S3). Indeed, across all years, winter social associations strongly predicted subsequent breeding proximity (Mantel r ~ 0.60; Fig. 1b). By carrying out permutations which re‐assigned individuals' social network positions within their winter spatial locations (i.e. the spatial null model), we found that winter spatial structuring contributed to the correlation between winter social networks and subsequent breeding proximity (Mantel r 0.44–0.52; Fig. 1b). However, as predicted, this relationship was significantly weaker than the observed carry‐over between prior social associations and future breeding positions (all years: *P* < 0.001, Fig. 1b). This demonstrates that the tightness of the social association between individuals related to how close their chosen breeding locations were to one another, over and above that expected from their winter spatial locations. This is particularly notable as the spatial null model was found to be highly conservative and generated social structure that was largely similar to the observed social networks (Fig. S4).

We found supporting results when the matrix denoting subsequent breeding proximity between individuals was regressed against matrices denoting (1) the winter social associations and (2) the winter dyadic spatial overlap, using MRQAP analysis. Individuals' winter spatial overlap significantly contributed to subsequent breeding proximity, yet even after accounting for this, the strength of fine‐scale social associations between dyads was a highly significant predictor of their breeding proximity (Table 1).

**Table 1:** Results of MRQAP tests. (a) Subsequent breeding season proximity (expressed as the reciprocal of Euclidean distance in metres) between individuals is significantly predicted by both their winter spatial overlap and their winter social associations prior to it. (b) Ranked version of proximity (to control for local breeding density) is also related to these two covariates

Response	Year	Covariate	Coefficient	*P*	Full *R* ^2^
(a) Euclidean distance	2012	Spatial overlap	0.0073	0.001	0.4310
		Social association	0.0106	0.001	
	2013	Spatial overlap	0.0062	0.001	0.4370
		Social association	0.0135	0.001	
	2014	Spatial overlap	0.0078	0.001	0.4126
		Social association	0.0037	0.001	
(b) Ranked proximity	2012	Spatial overlap	0.1203	0.001	0.3922
		Social association	0.3784	0.001	
	2013	Spatial overlap	0.1378	0.001	0.3971
		Social association	0.3340	0.001	
	2014	Spatial overlap	0.1727	0.001	0.3825
		Social association	0.2189	0.001	

We employed GLMMs to assess whether the positive relationship between winter social associations and subsequent breeding proximity may differ depending on the dyads considered. First, sex differences did not influence how winter social associations predicted breeding proximity, as there was no significant interaction between a factor indicating whether individuals were of the same or opposite sex and their winter social association strength on their subsequent breeding proximity (coefficient = − 0.001, *P*_null_ = 0.237 – Table S2a).

Second, we considered the influence of the previous year's breeding positions. In each breeding season, a small proportion of individuals had bred in the same location the previous year (~ 12%), and only ~ 0.5% of neighbours were neighbours the previous year due to remaining in the same locations. Nevertheless, in general, adult birds' breeding positions in the previous year were found to be related to the following winter's social associations and the subsequent year's breeding proximity (Table S3). However, when controlling for adults' breeding proximity to one another from the previous year, their social associations during the following winter remained a highly significant predictor of their subsequent breeding proximity (coefficient = 0.015, *P*_null_ < 0.001 – Table S2b). Similarly, for juvenile birds, the winter social associations formed in their first year predicted the proximity of their first breeding attempts (coefficient = 0.046, *P*_null_ < 0.001).

Finally, some individuals (~ 16%) foraged at a single feeding location all winter. By considering dyads that remained at the same, single, location as one another, we found that individuals subsequently bred closer to those they held stronger winter social associations to (coefficient = 0.033, *P*_null_ < 0.001 – Table S2c). Therefore, even within a single winter location, fine‐scale winter social association strength between dyads increased their subsequent breeding proximity.

Regardless of the type of individuals or dyads considered or methods used, it was consistently found that individuals subsequently bred closer to those they were most socially associated with during the winter, over and above that expected from their winter spatial locations (Table S2; Fig. S5).

#### Winter social networks shape breeding spatial arrangements

Along with subsequently breeding closer to their winter social associates, individuals could also arrange themselves so that they share breeding territory boundaries with those they had associated most with over the winter. Therefore, we tested whether individuals had held stronger winter social associations to birds they formed a breeding territory boundary with (i.e. their ‘neighbours’) than birds that bred the same distance away but with which they did not share a boundary (i.e. their ‘non‐neighbours’).

**Figure 2:**
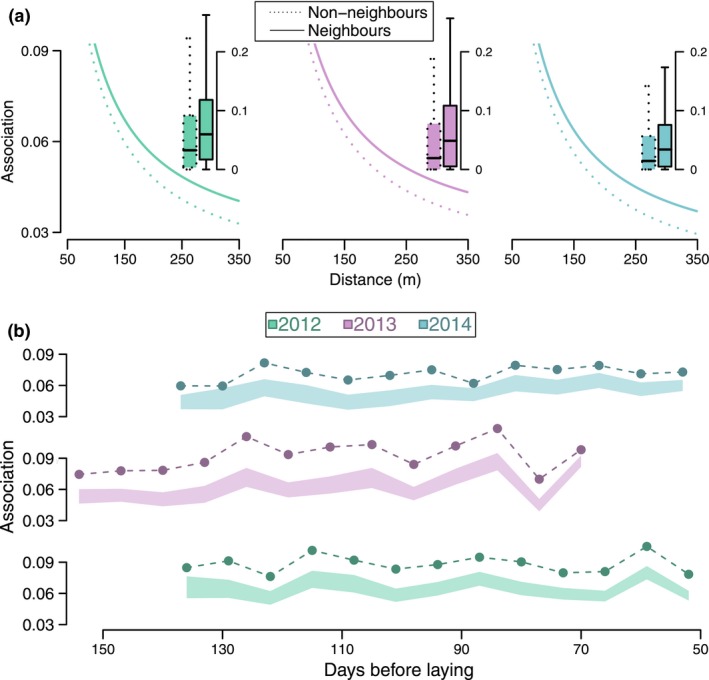
a) Relationship between winter social association strength and subsequent breeding distance for subsequent neighbours (solid lines) and non‐neighbours (dotted lines). Lines are based on the GLMM (see Table S4 for details) and colour denotes year. Box plots show each individual's winter social association to subsequent non‐neighbours (dotted – left) and neighbours (solid – right) within the range of their closest non‐neighbour and furthest neighbour (Fig. S2). Mid‐lines show median, box shows interquartile range (IQR), whiskers shows range (with values outside 1.5 times IQR excluded). b) The average winter social association strength individuals held to their subsequent neighbours at each sampling period (weekend) prior to it. Only social associations between subsequent neighbours are considered. *X*‐axis shows the number of days before the mean lay date in the following breeding season. Circles show the observed average winter association strength, and polygons show the 95% range calculated from the spatial null model.

We found that considering dyads that subsequently bred within the range to be neighbours or non‐neighbours (Fig. S2), there were significantly stronger winter social associations between neighbours than between non‐neighbours – even when controlling for the effect of winter social associations on breeding proximity (Fig. 2a). Thus, while individuals bred nearer to their closest winter associates, there was an additional effect of being more likely to share a territory boundary, and this was not driven by winter spatial locations (coefficient = 0.0075, SE = 0.002; *P*_null_ > 0.01; Fig. 2a; Table S4). Therefore, the boundary arrangements between territorial neighbours also reflected the prior winter social associations, more so than expected by breeding proximity or prior winter locations.

Indeed, winter social associations between subsequent breeding neighbours were particularly strong, and accounted for almost one‐third of the sum of all social associations over the entire winter social networks (2012 = 28%, 2013 = 32%, 2014 = 30%). Winter social associations between neighbours were also significantly stronger than expected from individuals' winter spatial positions, with a 1.7‐fold higher association strength than that generated by the spatial null model (Fig. 2b; Fig. S6). Strikingly, from early winter (even up to 150 days prior to the breeding season), individuals were more strongly socially associated to their subsequent breeding neighbours than expected under the spatial null model (Fig. 2b). This effect was maintained as the breeding season drew nearer, illustrating how temporally distant winter social association patterns can be important in predicting the subsequent spatial arrangement of individuals during breeding.

## Discussion

We demonstrate that prior social networks have trans‐seasonal carry‐over effects onto subsequent spatial structure within a wild bird population. Specifically, great tits' winter social associations, as they forage in freely moving flocks, are related to their long‐term spatial decisions regarding who they breed next to in the subsequent spring. By carrying‐over into future spatial structure, previous social structure may also be important to various processes that depend on individuals' locations and the environmental setting they experience. For instance in this study population, the relationship of winter social networks with subsequent breeding positions might have numerous consequences, as breeding structure is known to underpin both ecological (Garant *et al*. 2005; Cole *et al*. 2015; Hinks *et al*. 2015) and social processes (Grabowska‐Zhang *et al*. 2012a; Patrick *et al*. 2012; Firth *et al*. 2015a).

Individuals' spatial positions determine not only the environmental conditions they experience but also their social setting. Therefore, when these choices are fixed over a period important to reproductive success, individuals may benefit from choosing locations based on their previous social associations. For instance, maintaining stable social associations can promote reciprocation of cooperative interactions (Croft *et al*. 2006; Carter & Wilkinson 2013). Indeed, within breeding great tits, familiar birds are more likely to cooperate in predator mobbing (Grabowska‐Zhang *et al*. 2012a). Positioning oneself close to social associates may also reduce energy expenditure on competitive interactions, if individuals display less aggressive behaviours towards familiar neighbours (Temeles 1994). Breeding male birds, for example, appear to have more dissimilar song rates to surrounding individuals who they are more associated with (Foote *et al*. 2008; Snijders *et al*. 2015), potentially due to attempting to reduce competitive interference. Similarly, great tits may experience increased reproductive success under conditions of reduced competition with familiar neighbours (Grabowska‐Zhang *et al*. 2012b). Therefore, the potential benefits between close social associates may cause individuals to subsequently breed closer to (Fig. 1, Table 1), and share a territory boundary with (Fig. 2), those they were more strongly associated to during the winter.

While previous research has demonstrated that mated partners shape each other's foraging locations (Firth *et al*. 2015b), here we show that a link between sociality and spatial structure can become fixed over a long period and be driven by more subtle social relationships, as social associations to other flock members, even from early in the winter (Fig. 2b), predicted subsequent long‐term breeding locations. This finding may offer an explanation for why breeding location choices sometimes appear to be suboptimal in terms of the habitat (Chalfoun & Schmidt 2012), as such decisions may not be solely based on the features of the external environment, but also depend on the suitability of the fine‐scale social landscape. Further work that assesses how individuals balance environmental and social factors when making spatial choices, along with quantifying the relative fitness benefits of positioning themselves around their previous social associates, would now be particularly informative.

Breeding near prior social associates may also potentially result in increased matings between neighbouring individuals, as prior social associations may increase attractiveness (Choudhury & Black 1994). But, the relationship between winter social associations and subsequent breeding positions was not related to sex differences (Table S2a) and also occurred between same‐sex dyads (Fig. S5; Fig. S6). In fact, over all years and all classes of individuals (sex, age and movement patterns), winter social networks between individuals consistently predicted their subsequent breeding proximities and neighbours more than their winter spatial locations alone (Fig. S5; Fig. S6). For adult birds, winter social association predicted their future breeding proximity (Fig. S5; Fig. S6). This remained the case even when additionally controlling for their spatial positions in the previous breeding season (Table S2b), which can also influence winter social associations (Table S3). Similarly, birds that foraged in the same set location as one another all winter also subsequently bred closer to, and were more likely to form territory boundaries with, those they had held the strongest winter social connections with (Table S4; Fig. S5; Fig. S6). This suggests that the potential benefits of a trans‐seasonal carry‐over of previous social associations onto future spatial positioning are not only relatively broad but also strong enough that the effects are detected even after accounting for various individual and spatial factors.

Finding consistent and clear effects of winter social associations on subsequent breeding spatial arrangements after controlling for spatial factors is particularly striking when, as discussed in previous work (Shizuka *et al*. 2014; Aplin *et al*. 2015), an individuals' winter spatial location may also be driven by their social preferences. Disentangling social and spatial choices is challenging (Shizuka *et al*. 2014). However, we demonstrate a pronounced relationship between social associations and spatial decisions. Further, we provide this evidence in the particularly important context of spatial positioning of breeding attempts, where such decisions are fixed over a long and crucial time period, and the operation of various conspecific interactions, such as cooperation, competition and mating, depends on a single spatial choice.

In light of the evidence presented here, caution may be needed when accounting for spatial proximity in certain analyses. For example, if females of a certain type mate with males of a certain type, but also occur nearest to these individuals, controlling for distance might lead to a conclusion of no active mating choice, even if females' location choices are based on their preference for particular males. Through failing to recognise that observed spatial structure may itself be a product of individuals' social preferences, spatial terms fitted within a model may actually partly capture processes of interest.

Furthering the understanding of population spatial structure has various potential uses for conservation biology (Ferriere *et al*. 2004), and therefore, the findings of this study may have practical implications. For instance, playing conspecifics' calls is a conservation strategy used to attract target bird species to particular breeding sites when attempting to minimise adverse effects of anthropogenic activity (Ward & Schlossberg 2004; Ahlering & Faaborg 2006). However, if individuals choose to breed near their previous social associates, simulating conspecific calls that are likely to be familiar may further increase the effectiveness of this approach. Indeed, increasing knowledge of nesting‐site decisions is beneficial for applied work aiming to improving avian species population viability (Chalfoun & Schmidt 2012).

To conclude, we demonstrate that spatial structure during breeding, when individuals make a fixed decision on a long‐term location, is underpinned by prior social associations occurring as individuals take part in loose, freely moving, foraging flocks over winter. Prior social networks may also, therefore, carry‐over into various population‐level processes through shaping individuals' future environmental and social settings. Further research that directly manipulates social behaviour (Firth & Sheldon 2015) to assess the extent of the causal effect of social associations on subsequent breeding locations would be valuable. Indeed, revealing the long‐term benefits and causal implications of holding social associations is of vital importance for elucidating the underlying drivers of social dynamics.

## Authorship

JAF and BCS designed the study and contributed to data collection. JAF carried out the analysis and wrote the first draft and BCS contributed substantially to revisions.
