# Supplementary material for: Social carry‐over effects underpin trans‐seasonally linked structure in a wild bird population
Source: Ecol Lett. 2016 Sep 13;19(11):1324–32. doi: 10.1111/ele.12669 (PMC5082527; doi:10.1111/ele.12669)
Supplement: Supplementary file 1 [file ELE-19-1324-s001.docx]

**SUPPLEMENTARY INFORMATION: Social carry-over effects underpin trans-seasonally linked structure in a wild bird population**

*Josh A. Firth^1^ & Ben C. Sheldon^1^*

*^1^Edward Grey Institute, Department of Zoology, University of Oxford, Oxford, OX1 3PS, UK*

**Supplementary Information – Additional Details:**

(a) Spatial null model

As social data are inherently non-independent, null models are widely utilised in animal social network analysis (Whitehead 2008; Croft *et al.* 2011). In particularly, null models are often used for separating out the contribution of the social preferences and the spatial distribution of individuals (Whitehead 2008; Shizuka et al. 2014; Aplin et al. 2015; Farine et al. 2015).

We aimed to assess how individuals’ winter social associations related to their subsequent breeding proximity to one another. However, as the study was carried out over a large spatial scale, it is likely that the spatial location of an individual during the winter will influence their winter social associations (as they form flocks with individuals in the same area) and their breeding position (as we would not expect a random redistributing of individuals after the winter). Specifically, if individuals remain in their same location at the end of the winter for breeding, this may cause them to breed close to others who also occurred there during the winter. In turn, this would result in individuals breeding near to those they were likely to have formed winter flocks with, and therefore a relationship between winter social associations and subsequent breeding positions would arise due to individuals’ spatial locations/preferences.

Therefore, we used a null model to determine the extent of the relationship between winter social associations and subsequent breeding positions that may be expected under this spatial process alone (Fig. S1). If the observed relationship between winter social associations and subsequent breeding positions was significantly larger than the one generated by this spatial process alone, we can conclude that the relationship is not just driven by winter spatial distribution of individuals, and therefore this suggests that active social preferences are contributing to this relationship.

Each permutation of the null model began by assigning each individual to the winter location (feeding station) they were last observed before the breeding season began (89% of individuals were observed in the final sampling period, 98% in the final month). For individuals observed in multiple locations during their last period (45%), in each permutation, the probability of assignment to each location was equal to their activity (proportion of flocks they were observed in) at each feeding station. Positions of individuals within the social network (which are equivalent to the node labels on the social association matrix) were then swapped so that each adopted the identity of another individual with their same assigned winter spatial location (Fig. S1b). A similar permutation process has been used in previous work in this study system (Aplin et al. 2015) to generate permutated social structure whilst controlling for individuals’ spatial locations. An advantage of this method, over permuting the raw data, is that the network structure (i.e. actual dyadic values of social association between individuals) remains the same but the identity of individuals’ is swapped within spatial locations. Thus, each permutation maintains the network structure and spatial location of individuals, but reassigns the dyadic social associations given these constraints (Fig. S1).

In this way, after we had calculated a statistic of interest assessing the relationship between the observed winter social associations with the subsequent breeding structure (whether this was a mantel test correlation, or a coefficient of a GLMM – see Main Text), we could then carry out the same statistical test 1000 times, but each time replacing the observed winter social associations with one of the 1000 versions of the permuted winter social associations. This generated the distribution of the statistic assessing the relationship between winter social associations and subsequent breeding structure expected under the spatial null model. If the statistic derived from using the observed winter social associations fell outside of the 95% range of the distribution of the null model, it can be inferred that there is a significant effect of fine-scale winter social associations patterns on subsequent breeding positioning, on top of that expected from winter spatial factors and general network structure alone.

As explained above, in each permutation of the null model, the individuals were reassigned to adopt the association patterns of others occurring the same final winter foraging locations as themselves *at random* (Fig. S1). However, some of our analysis specifically considered how the relationship between winter social associations and subsequent breeding positions may differ between individuals of different classes (sex, age, movement patterns). Therefore, in these analysis, in order to control for individual characteristics, we also carried out various versions of the same null model. Again, individuals could only be reassigned to the social association patterns of others in the same winter foraging locations, but with the added restriction that they could only be assigned to those of the same ‘class’ (i.e. whichever trait was considered) as themselves.

First, we only allowed reassignments within the same sex, thereby examining the males and females separately, as well as reducing any issues arising from non-independence of pair members. Second, swaps were restricted within age classes to enable first-year birds, with no associations from previous breeding seasons, to be assessed separately from adults. Third, to control for individual movement patterns, we carried out swaps between birds that remained at one feeder throughout the winter (termed ‘set’ birds, which may be expected to hold strong associations with others who remained there), and then between birds that moved locations (termed ‘moving’ birds).

Finally, as stated previously, the primary null model considers the final winter locations of where each individual was observed, as this is expected to be most strongly related to where they breed, and therefore more conservative method of controlling for spatial factors. However, as part of the supplementary analysis we also carried out the same null model but instead considering the entire winter range of each individual. The process was again the same, but in each permutation each individual was assigned to one of the locations where they were observed over the entire winter (rather than just during their final observation period). Again, the probability of assignment to each location was equal to their activity (proportion of flocks they were observed in) at each feeding station. In this way, in each permutation individuals were most likely to ultimately be reassigned the dyadic social associations of another individual who most often occurred in the same location as themselves (see Aplin et al. 2015). This, however, was shown to be similar to the primary null model considering the final winter locations of individuals (Fig. S4).

(b) Matrix Regression

Multiple Regression Quadratic Assignment Procedure (MRQAP) is a useful technique in network analysis as it allows multiple independent matrices (i.e. x_1_ and x_2_) to be simultaneously regressed against a single dependent matrix (y_1_). In particular, an extension of MRQAP, termed MRQAP with double semi-partialing (MRQAPDSP), allows increased inference of the relationship that y_1_ holds with both x_1_ and x_2_ separately, even when there is strong multicollinearity between the fixed effects (Dekker et al. 2007). For this reason, this is a useful approach for assessing the separate effect of winter social structure and winter spatial overlap (which are often highly correlated) on a single independent network (Shizuka et al. 2014; Firth & Sheldon 2015).

Therefore, in this work, we were able to assess how subsequent breeding proximity (i.e. ‘y’) was related to the prior winter social network (‘x_1_’) given the effect of winter spatial overlap between individuals (‘x_2_’). To determine the ‘winter spatial overlap’ of between dyads, we took a conservative approach and aimed to consider both the amount of range overlap of each dyad, as well as the amount of activity across their range. Therefore, we first computed the proportion of activity each individual spent at each location. Then, the winter spatial overlap for each dyad was determined by calculating the minimum proportion of activity that either member spent at each location, and then taking the sum of these values over all locations. In this way, the value for each dyad shows both how much their spatial ranges overlapped, as well as how much their activity within their ranges overlapped. Indeed, the scale ranges from 0 to 1, where dyads with identical proportions of activity at each place to one another score 1, those that never overlapped score 0, and those that overall spent half of their activity in the same locations as one another would score 0.5. For instance, when using this method, birds (B_1_ & B_2_) who only occurred at the same two feeding stations (F_1_ and F_2_) as one another, with B_1_ spending 90% of their time at F_1_, and B_2_ spending 90% of their time at F_2_, would score 0.2 as their winter spatial overlap i.e. 0.1 (F1 minimum activity) + 0.1 (F2 minimum activity). On the other hand, if B_2_ had also spent 90% of their activity at F_1_, they would score 1 i.e. 0.9 (F1 minimum activity) + 0.1 (F2 minimum activity).

Thus, using MRQAPDSP, we used i) this winter spatial overlap matrix, and ii) the winter social network, as predictor matrices and simultaneously regressed them over the breeding proximity matrix. This assessed whether the social network was a significant predictor of subsequent breeding proximity given individuals’ winter spatial overlap. As this considers individuals’ winter spatial overlap over the entire winter, it is distinctly different from the primary null model analysis that considers that individuals are likely to remain in the same area at the end of the winter to breed. However, we also ran a modified version of the null model aiming to consider individuals locations over the entire winter that produced the same patterns (see above and Fig. S4).

**Supplementary Figures**

**Figure S1**. Graphical depiction of the analytical procedure using computer-generated example data. The large numbers show four different feeding stations, and surrounding circles (of the same colour) show the individuals who occurred there in the winter before the breeding season began. Letters indicates unique identification code of the individual, and connecting lines (‘edges’) show social associations occurring between them whilst the thickness of lines show the strength of the social association. a) The observed winter social network generated from the flocking co-occurrences between individuals over the winter. b) Example of one permuted social network after the null model has been carried out, whereby individuals at the same location are reassigned social network positions within this location whilst social network structure is held constant. c) The individuals’ observed breeding positions, where individuals are generally likely to breed relatively close to their winter foraging location, but the proximity of individuals breeding attempts to one another appears more strongly related to their observed prior winter social associations (a), than to their permuted winter social associations (expected from their winter foraging locations alone) (b).

**Figure S2.** Example map showing territories around individuals’ breeding locations. Bird icon shows the breeding location of the focal individual, and dots show other individuals’ breeding locations. The black lines show the inferred territory boundaries around each individuals breeding location (derived using voronoi polygons – see Methods). Those who share a territory boundary are classified as neighbours, those who do not share a territory boundary are classed as non-neighbours. The focal individual’s nearest non-neighbour and the furthest neighbour are shown in bright red. The boundary between the nearest non-neighbour and further neighbour is shown in red. Only individuals’ social associations to those who subsequently fell within this boundary were considered when assessing whether individuals held stronger winter associations to their subsequent breeding neighbours than to their subsequent non-neighbours (see Methods, Fig. 2 & Table S4). Therefore, only dyadic links between those who subsequently bred within a given proximity to be either neighbours or non-neighbours was considered. In this way, it was not the case that proximity was strongly related to whether or not individuals shared a territory boundary. Indeed, this method meant that each individual’s nearest individual considered was then a non-neighbour, and the furthest one was a neighbour. This also had the beneficial consequence of reducing multicollinearity for the model including fixed effects of breeding proximity as well as whether or not individuals were neighbours.

**Figure S3**. Summary figure showing the decline in dyadic winter social associations over subsequent breeding proximity when measured as a) distance in metres, beginning at 60m (average distance to nearest individual) at continuing in bins of 20m (average step distance to continuing next nearest individuals) and b) Ranked closest individuals. Vertical lines show standard error around mean. Colour indicates year. Even at relatively fine spatial resolutions, stronger winter social associations related to increased proximity in the subsequent breeding season


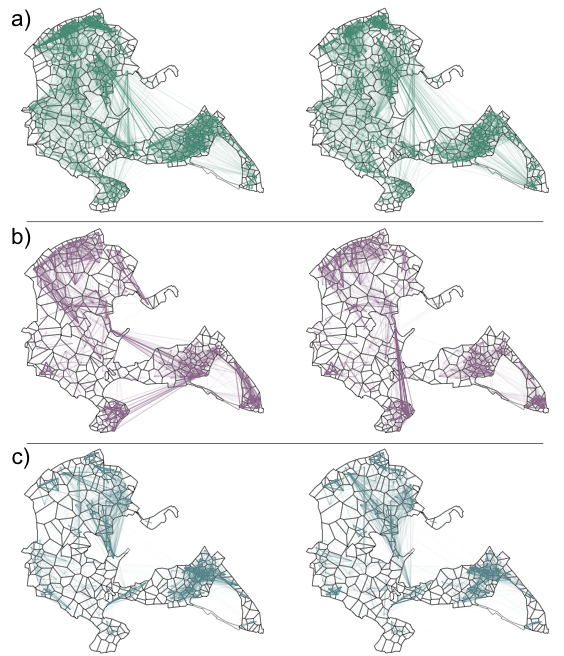


**Figure S4.** Comparison of the observed social networks (left hand side) with a social network generated from the null model permutations (right hand side). Winters ending in a) 2012, b) 2013 and c) 2014 are shown. Surrounding border shows Wytham woods, and each individual is in the location where they were observed breeding (points not plotted for clarity). Connecting lines show winter social associations (thickness represents strength). In cases where both the male and the female at a particular box where associated with another individual at another box, the mean strength is displayed. The observed networks were highly structurally similar to the networks generated from the permutation process: Mantel r (mean and 95% range) = 2011: 0.62 (0.59-0.66), 2012: 0.64 (0.60-0.70), 2013: 0.64 (0.59-0.70). Further, networks generated from the permutation process but using individuals’ feeding locations over the entire year (instead of their final location) were also highly similar to observed networks: Mantel r (mean and 95% range) = 2011: 0.61 (0.56-0.66), 2012: 0.68 (0.63-0.73), 2013: 0.65 (0.59-0.71).

**Figure S5.** Mantel r test statistic assessing the relationship between winter social networks and subsequent breeding proximity matrix when links between dyads of particular classes are considered separately. Proximity was measured using a) Euclidean distance and b) ranked nearest individuals. Symbols show the observed statistic, and boxes show the 95% range of statistics calculated from the spatial null model that controlled for class-type. Mid-lines illustrate mean of the statistics from the null model. X axis shows denotes which dyadic links were considered. “M-F” = intersexual links only, “FY-Ad” = between age classes only, “Move-set” = between those that moved during the winter (“moving”) and those that remained in the same location (“set”). The effect, in terms of the correlation between the observed winter social network and the breeding proximity compared to the correlation generated from using the permuted social networks, was similar across classes. The only exception was in 2013 when winter associations between first-year birds did not predict their breeding proximity more than expected from the null model. This is most likely to have been driven by the small sample size of first-year birds due to adverse weather conditions causing high mortality of nestlings in the previous year (only 17.4% of breeding individuals recorded in this winter were first-year birds – Table S1). Indeed, on a smaller scale, social associations between subsequent neighbours were found to be stronger than expected from their winter spatial distribution (see Fig. S6).

**Figure S6**. Individuals’ average winter social association strength to their neighbours in the subsequent breeding season. Only social associations between neighbours are considered. Symbols show the observed mean with vertical lines showing standard error. Boxes show the 95% range of statistics calculated from the spatial null model which controlled for class-type. Mid-lines illustrate mean of the statistics from the null model. X axis shows denotes which dyadic links were considered. “M-F” = intersexual links only, “FY-Ad” = between age classes only, “Move-set” = between those that moved during the winter (“moving”) and those that remained in the same location (“set”). Hence, even within sexes, within age classes, and within individuals that moved and did not, as well as between all these classes, individuals were more strongly associated in the winter with those they subsequently shared territory boundaries with than expected from their winter spatial locations.

| Split | Class | Individuals | | | Social network links | | | Neighbour links | | |
| --- | --- | --- | --- | --- | --- | --- | --- | --- | --- | --- |
|  |  | **2011** | **2012** | **2013** | **2011** | **2012** | **2013** | **2011** | **2012** | **2013** |
| - | All | 396 | 272 | 247 | 7909 | 3085 | 2880 | 922 | 686 | 519 |
| Sex | Male | 163 | 137 | 119 | 1236 | 834 | 672 | 151 | 193 | 126 |
|  | Female | 233 | 135 | 128 | 2902 | 727 | 761 | 323 | 156 | 136 |
|  | M-F | - | - | - | 4138 | 1561 | 1433 | 474 | 349 | 262 |
| Age | First-year | 179 | 47 | 137 | 1797 | 97 | 900 | 179 | 24 | 171 |
|  | Adult | 217 | 225 | 110 | 2102 | 2035 | 549 | 272 | 459 | 83 |
|  | FY-Ad | 0 | 0 | 0 | 3899 | 2132 | 1449 | 451 | 483 | 254 |
| Movement | Moving | 342 | 214 | 212 | 6732 | 2216 | 2490 | 724 | 473 | 412 |
|  | Set | 54 | 58 | 35 | 70 | 102 | 20 | 41 | 32 | 8 |
|  | Move-Set | - | - | - | 6802 | 2318 | 2510 | 765 | 505 | 420 |

**Supplementary Tables**

**Table S1**. Sample sizes and dyadic links between individuals who occurred in the social network and also were recorded in the subsequent breeding season. ‘Individuals’ columns show the counts of those in each demographic class each year, ‘social network links’ columns show the number of social network edges between those in that class that year, and ‘neighbour links’ columns show the number of breeding season neighbour boundaries shared. Hyphenated classes show ‘between-class’ counts.

**Table S2.** Output of GLMMs assessing the relationship between winter social associations and subsequent breeding proximity for different classes of individuals. In all models, subsequent breeding proximity (reciprocal of Euclidean distance) between each dyad was the dependent variable. All models included winter social association strength and year as predictor variables. Random effects of both individuals’ identities and their nest boxes were also included. Model a) included a factor indicating whether each dyad was same-sex (scored as 1) or opposite-sex (scored as 0), as an additional predictor variable, along with its interaction with winter social association. Model b) excluded all individuals except adults who bred the previous year, and included a predictor variable denoting the previous breeding proximity between these adults before the winter began. Model c) excluded all dyadic links except from those between individuals who remained in the same single location as one another over the winter. The p-value_null_ shows the p-value for the coefficient generated from comparison to the coefficients from the permutations under the spatial null model.

| Model | Covariate | Coefficient | SE | t -value | p-value_null_ |
| --- | --- | --- | --- | --- | --- |
| A | Association | 0.05122 | 0.00024 | 209.46244 | **0.001** |
|  | Sex | 0.0001 | 0.00001 | 8.71265 | **0.015** |
|  | Sex*Association | -0.00116 | 0.00035 | -3.34968 | 0.237 |
|  | Year 2012 | -0.00006 | 0.00002 | -2.71562 | 0.741 |
|  | Year 2013 | -0.00007 | 0.00005 | -2.43136 | 0.326 |
| B | Association | 0.01537 | 0.0004 | 38.46216 | **0.001** |
|  | Prev. Proximity | 0.63118 | 0.00466 | 135.35404 | **0.001** |
|  | Year 2012 | -0.00002 | 0.00002 | -0.8072 | 0.206 |
|  | Year 2013 | 0.00005 | 0.00005 | 0.48479 | 0.654 |
| C | Association | 0.03303 | 0.00944 | 3.50026 | **0.001** |
|  | Year 2012 | -0.00092 | 0.00117 | -0.78374 | 0.746 |
|  | Year 2013 | -0.00299 | 0.00158 | -1.88731 | 0.946 |

**Table S3.** Results of MRQAP tests assessing the relationship between adult birds’ breeding proximities to one another in the previous year to the following year winter social network and the subsequent year’s breeding proximity. Only birds that were recorded in all three periods were considered (n=125, 148 and 85 for 2012, 2013 and 2014 respectively).

| Year | Covariate | Coeff. | P | Full R^2^ |
| --- | --- | --- | --- | --- |
| 2012 | Subsequent Social Association | 0.0111 | 0.001 | 0.6563 |
|  | Subsequent Breeding Proximity | 0.7955 | 0.001 |  |
| 2013 | Subsequent Social Association | 0.0222 | 0.001 | 0.6767 |
|  | Subsequent Breeding Proximity | 0.6364 | 0.001 |  |
| 2014 | Subsequent Social Association | 0.0113 | 0.001 | 0.7051 |
|  | Subsequent Breeding Proximity | 0.9239 | 0.001 |  |

**Table S4**. Output of GLMMs assessing the relationship of winter social associations with subsequent breeding proximity and subsequent breeding territory boundary sharing (whether individuals were breeding neighbours or not) (Fig. 2a). Only dyadic links that were within the range of their closest non-neighbour and furthest neighbour were considered (Fig. S2). Year was also included as fixed effect, whilst both individuals’ identities and both their nest boxes were set as random effects. The p-value_null_ shows the p-value for the coefficient generated from comparison to the coefficients from the permutations under the null model.

|  | Coefficient | SE | t -value | p-value_null_ |
| --- | --- | --- | --- | --- |
| Intercept | 0.013 | 0.004 | 3.2067 | 0.294 |
| Proximity | 7.0395 | 0.4723 | 14.9034 | **0.01** |
| Neighbour | 0.0075 | 0.002 | 3.6906 | **0.01** |
| Year 2013 | 0.0029 | 0.0033 | 0.8644 | 0.626 |
| Year 2014 | -0.0034 | 0.0036 | -0.946 | 0.558 |
